# Annotations

**Published:** 1890-01-04

**Authors:** 


### Annotations.

**Influenza, Scarlet Fever, and Dengue.**

A contemporary heads an annotation, "Dengue or Influenza," and remarks that an interesting feature of the prevailing Russian epidemic, which has attracted some attention in Paris, is the variety of symptoms which the present epidemic of influenza has exhibited from those observed previously. In some cases the predominant feature has been muscular pains, and in some instances a scarlatiniform eruption. The question of the identity of the two maladies, dengue and influenza, has been recently discussed in the Paris Academy of Medicine, and, as usual among doctors, some difference of opinion prevailed. Dr. Felkin, in his recent article "On the Geographical Distribution of Some Tropical Diseases" (*vide* "Hospital Retrospect," November 23), limits the area of dengue to India, Arabia, the African coast of the Red Sea, the coast of the Carribean Sea, the West Indies, and the Southern States of America. As to the specific nature of dengue there can be little doubt that we know as little of it as of the specific nature of influenza. For, as pointed out in the "Hospital Retrospect" of December 28th last, the symptoms of a common ordinary coryza are the ordinary symptoms of influenza. The symptoms of dengue, however, are different. In the greater number of cases the first symptoms of dengue are headache, restlessness, chilliness, pains in the back, limbs, eyelids, and joints. But sometimes there are no preceding feelings of malaise, the pains and fever commencing quite suddenly. In about twelve hours, or exceptionally after a longer period, an eruption of a red or scarlet character appears, lasting about forty-eight hours, and there is generally sore throat. The glands of the neck especially may also be swollen. With the decline of the eruption the fever subsides, and there is almost total remission. But most frequently, after three or four days, eruption and fever again return, but this second eruption more resembles nettlerash than scarlet fever. The subsidence of fever and the disappearance of the eruption often leave the patient much weakened, with rheumatic soreness and stiffness of the joints. Albuminous urine may be a sequence, but this gradually disappears. It will thus be seen that dengue more resembles scarlet fever than influenza, although there are broad distinctions between the two maladies. Dengue occurs in a more decidedly epidemic manner than scarlet fever. A high temperature is more rapidly attained in dengue than in scarlet fever, and it declines more rapidly. The severe muscular and joint pains of dengue are not characteristic of scarlet fever. Dengue does not confer protection against recurrence. The character of the secondary eruption generally differs. Still it is probable that dengue and scarlet fever are allied diseases; more so than it would seem dengue and influenza are allied. In considering the question of the identity of diseases, temperament, constitution, and idiosyncrasy should be taken into account more than is usually done. All diseases vary from their typical portrait. Scarcely any two cases of the same disease are precisely similar. Diseases cannot be judged of from the experience of one patient, of one locality, of one epidemic, of one country, or even of one zone. In many instances a disease in a particular patient may but present a blurred image of the type. Various articles of food and many medicines produce different effects on different people, for which we cannot account, and are obliged to refer them to peculiarities of constitution, of temperament, to idiosyncrasy, and to environment, which, as is well known, may modify the course of a disease. We do not discern any reason for regarding the present influenza epidemic as dengue, nor for elevating the latter from its position as an exceptionally severe catarrh, or popularly a "bad cold."

**The Drawing-room and the Epidemic.**

It seems probable that in the course of two or three thousand years the cerebrum of the average woman of the British middle class will be so far developed as to enable her to make a proper use of her drawing-room in cold, damp weather. We ourselves use strenuous efforts to further this desirable end, and, thanks to the sustaining power of the Darwinian faith, we are able to keep alive a reasonable hope that future generations will enjoy the fruits of our disinterested labours in this direction. The epidemic of influenza now prevailing on the Continent enables us to point a moral if it does not add much to our medical knowledge. The real cause of the epidemic seems to be the prolonged damp weather, associated with cold. The persons who suffer most from it are those who live indoor lives and take little physical exercise. People of this class may resist cold weather if it be dry, and damp weather if it be warm; but cold and damp together form a combination which is dangerous or even fatal. But what connection has the middle-class British housewife and her drawing-room with the influenza epidemic? This—that she occasionally uses that sacred apartment, and uses it in such a way as to make it, if not a death-trap, at least an influenza or a pneumonia trap for all but the very strongest. She will, for instance, invite her friends to a dinner party which may fall on one of the coldest and dampest days of the winter. She will have the drawing-room fire lighted exactly half-an-hour before her guests begin to arrive. When it is remembered that many drawing-rooms have fires in them only once a week, or even far less frequently, it is seen at once that a room full of damp air and enclosed by damp walls cannot even begin to be warm in half-an-hour. Nay, more, it cannot be anything like thoroughly warmed in two or three hours. When the guests arrive, the men in thin black clothes, and with chests almost unclothed; the ladies in evening dress, which is equivalent to shoulders and chests quite bare, they find that the drawing-room in which they assemble is little better than an ice-house. Even when the dinner is over and they have trooped back from the dining-room, those who cannot get near the fire sit in chill draughts and damp currents that are almost as dangerous as a pestilence. The average man who thinks on these things is absolutely dismayed by the complete destitution of intelligence and forethought displayed by the good British housewife. What can be done, he says to himself in despair, to make the well-intentioned but desperately dull-witted woman see that if the drawing-room fire could but be lighted in the morning, when the other fires are lighted, and kept burning brightly all day, everybody would sit with a sense of comfort, no one would anathematise the miserable damps and draughts, and no one would have to pay a doctor's or an undertaker's bill as the result of a particular party? The writer would feel that he had not lived in vain if he could convert the average British housewife to the elementary but not unimportant religion of well-aired, well-warmed, and comfortable drawing-rooms. The conclusion that a very large number of competent observers have arrived at in this country is, that the epidemic is only an epidemic in the sense of its having attacked a very large number of persons, and not in the sense of being in any high degree contagious. In consequence of the decidedly peculiar weather a larger than ordinary number of people are having "bad colds," and those colds are unusually severe. There is, therefore, all the more reason why unusual care should be taken to keep rooms warm and dry.

